# Spontaneous Necker-cube reversals may not be that spontaneous

**DOI:** 10.3389/fnhum.2023.1179081

**Published:** 2023-05-30

**Authors:** Mareike Wilson, Lukas Hecker, Ellen Joos, Ad Aertsen, Ludger Tebartz van Elst, Jürgen Kornmeier

**Affiliations:** ^1^Department of Psychiatry and Psychotherapy, Medical Center – University of Freiburg, Freiburg, Germany; ^2^Faculty of Medicine, University of Freiburg, Freiburg, Germany; ^3^Institute for Frontier Areas of Psychology and Mental Health, Freiburg, Germany; ^4^Faculty of Biology, University of Freiburg, Freiburg, Germany; ^5^Department of Psychosomatic Medicine and Psychotherapy, Medical Center – University of Freiburg, Freiburg, Germany; ^6^INSERM U1114, Cognitive Neuropsychology and Pathophysiology of Schizophrenia, Strasbourg, France; ^7^Bernstein Center Freiburg, University of Freiburg, Freiburg, Germany

**Keywords:** ambiguous figures, perceptual reversals, bistable perception, EEG, Necker cube

## Abstract

**Introduction:**

During observation of the ambiguous Necker cube, our perception suddenly reverses between two about equally possible 3D interpretations. During passive observation, perceptual reversals seem to be sudden and spontaneous. A number of theoretical approaches postulate destabilization of neural representations as a pre-condition for reversals of ambiguous figures. In the current study, we focused on possible Electroencephalogram (EEG) correlates of perceptual destabilization, that may allow prediction of an upcoming perceptual reversal.

**Methods:**

We presented ambiguous Necker cube stimuli in an onset-paradigm and investigated the neural processes underlying endogenous reversals as compared to perceptual stability across two consecutive stimulus presentations. In a separate experimental condition, disambiguated cube variants were alternated randomly, to exogenously induce perceptual reversals. We compared the EEG immediately before and during endogenous Necker cube reversals with corresponding time windows during exogenously induced perceptual reversals of disambiguated cube variants.

**Results:**

For the ambiguous Necker cube stimuli, we found the earliest differences in the EEG between reversal trials and stability trials already 1 s before a reversal occurred, at bilateral parietal electrodes. The traces remained similar until approximately 1100 ms before a perceived reversal, became maximally different at around 890 ms (*p* = 7.59 × 10^–6^, Cohen’s *d* = 1.35) and remained different until shortly before offset of the stimulus preceding the reversal. No such patterns were found in the case of disambiguated cube variants.

**Discussion:**

The identified EEG effects may reflect destabilized states of neural representations, related to destabilized perceptual states preceding a perceptual reversal. They further indicate that spontaneous Necker cube reversals are most probably not as spontaneous as generally thought. Rather, the destabilization may occur over a longer time scale, at least 1 s before a reversal event, despite the reversal event as such being perceived as spontaneous by the viewer.

## 1. Introduction

During observation of an ambiguous figure, like the famous Necker cube (Necker, 1832), our perception becomes unstable, and alternates between two, or even more, possible interpretations despite unchanged visual input. Such perceptual reversals can occur spontaneously or suddenly, i.e., without the observer’s expectation or intention to perceive the sensory input in a different way. In contrast, observers can also volitionally control their percept to some degree and both increase and decrease their reversal rate (the number of perceptual reversals over time), however, they cannot prevent the reversals entirely (e.g., Strüber and Stadler, 1999; van Ee et al., 2005; Kornmeier et al., 2009, 2019). Multistable perception can also be induced via binocular rivalry, i.e., if the two eyes simultaneously receive conflicting information. Similar to the situation with classical ambiguous figures, perception alternates between the two percepts corresponding to the two eyes’ input (e.g., Blake, 2001; O’Shea et al., 2013). Multistable perception phenomena have also been reported in other modalities, like audition (e.g., Pressnitzer and Hupe, 2006; Einhäuser et al., 2020) and touch (e.g., Carter et al., 2008; Conrad et al., 2012; Liaci et al., 2016; Hense et al., 2019; Darki and Rankin, 2020).

The phenomenon of multistable perception has fascinated researchers from different disciplines for more than 200 years (Brascamp et al., 2018). Multistable perception is vividly discussed in the contexts of perception and consciousness (e.g., Crick and Koch, 1998; Blake and Logothetis, 2002; Long and Toppino, 2004; Atmanspacher et al., 2008; Giles et al., 2016; Brascamp et al., 2018; Devia et al., 2022), particularly because a separation of sensory processing (which can be kept constant over time) and perceptual processing (which alternates repeatedly) appears to be possible (e.g., Crick and Koch, 1998; Tong et al., 1998; Leopold and Logothetis, 1999; Intaite et al., 2010; Blake et al., 2014; Bartels, 2021). However, despite a large number of experimental studies, the neural mechanisms underlying spontaneous perceptual reversals are so far poorly understood. Problems are both theoretical and practical in nature. Very often, it is unclear what the processes are that (1) necessarily precede a spontaneous perceptual reversal (e.g., the weakening or destabilization of a neural representation) and causally lead to it, and (2) those of the reversal process as such, i.e., the change in dominance (and access to consciousness) of one neural representation by the alternative one in a certain time window, and finally (3) the subsequent processes, related to becoming aware of the reversal event and communicating it with the environment within an experimental paradigm (Brascamp et al., 2018; Devia et al., 2022). The Electroencephalogram (EEG) provides the necessary temporal resolution in the range of milliseconds to differ between these processing steps. However, the precise temporal reference of the reversal event is necessary for the differentiation between the above-described processing steps but at the same time difficult to access due to its endogenous nature.

A number of EEG- and fMRI-studies used participants’ key presses as a time reference for the reversal event (e.g., Basar-Eroglu et al., 1996; Lumer et al., 1998; Sterzer and Kleinschmidt, 2007; Knapen et al., 2011). This method, however, comes with the problem of intra-individual reaction time variability in the range of ± 100 ms (Strüber and Herrmann, 2002; Kornmeier and Bach, 2004, 2012), which is too large to exploit the high temporal resolution of the EEG. An alternative approach used ambiguous motion stimuli with specific stimulus features or presentation modes that allow to narrow down a possible time window of the reversal event (e.g., Pastukhov and Braun, 2013; Pastukhov, 2016; Weilnhammer et al., 2017, 2021). This elegant stimulus design, however, is restricted to a certain class of motion stimuli. De Jong et al. (2020) recently developed a clever method using within-participant intracranial electrophysiological measures to temporally resolve reversals of binocular rivalry stimuli.

Other studies referred to the change in pupil diameter during perceptual reversals (Einhäuser et al., 2008; Hupe et al., 2009; Kloosterman et al., 2015; Brascamp et al., 2021) or used the horizontal eye fixation positions in a free observation paradigm (Polgári et al., 2020). These innovative approaches allowed the implementation of “no report paradigms” and helped to separate task-related and perception-related processing steps (e.g., Brascamp et al., 2018, 2021; Devia et al., 2022). However, the specific role of the pupil response in the reversal process is discussed controversially (Duman et al., 2022). It is particularly unclear, whether the pupil response or a certain change in horizontal eye position precedes or follows the reversal event and how much the latency between the reversal event and the pupil response/eye position change varies across repetitions.

In the present study, we used the so-called “Onset Paradigm” to estimate the timing of the reversal event. The basic idea goes back to the seminal work of Orbach et al. (1963, 1966). They presented the Necker cube discontinuously and found a huge variability of the reversal rate (number of reversals per minute) as a function of the inter-stimulus interval (ISI), i.e., the duration of a blank screen between successive Necker cube presentations. The reversal rate continuously increased from continuous presentations to ISIs of about 400 ms and decreased again for longer ISIs, up to a complete freezing of the percept for ISIs of about 2 s (Leopold et al., 2002; Maier et al., 2003; Kornmeier et al., 2007; Pastukhov and Braun, 2008; Pearson and Brascamp, 2008; Zaretskaya et al., 2010). O’Donnell et al. (1988) were the first to present Necker cubes discontinuously while measuring EEG. They found a P300 event-related potential (ERP) component correlating with a perceptual reversal. Kornmeier and Bach (2004) adopted this idea and developed the “Onset Paradigm”: Participants compared their percepts of subsequently presented Necker cubes and indicated in separate experimental conditions either perceptual reversals (reversal condition) or perceptual stability (stability conditions) from the previously observed stimulus to the current stimulus by a key press in the subsequent ISI. Such Necker cube reversals were labeled “endogenous” because the Necker cube as such is equally compatible with both 3D interpretations and the perceptual reversal cannot be related to certain stimulus features. The idea of this paradigm was, that the discontinuous presentation mode synchronizes the reversal event with stimulus onset, allowing to use the latter as a time reference for the former. Kornmeier et al. calculated ERPs and subtracted stability trials from reversal trials in order to eliminate the ERP signatures related to stimulus onset, decision making, and motor preparation.

In a separate experiment with the same conditions, Kornmeier et al. replaced the ambiguous Necker cube with disambiguated cube versions, corresponding to the two perceptual interpretations of the ambiguous Necker cube stimulus. They labeled perceptual reversals evoked by the disambiguated stimuli as exogenous reversals. Their Onset Paradigm increased the temporal resolution of the reversal event strongly and revealed two highly similar chains of ERP components for endogenous perceptual reversals (Experiment 1) and exogenously induced reversals (Experiment 2) with two exceptions. The ERP chain related to the endogenous reversals started with a small occipital positivity (“Reversal Positivity”) at 160 ms after stimulus onset. This Reversal Positivity was not observed with exogenously induced reversals. It may indicate an early visual “detection” of a maximally unstable perceptual state in the case of an endogenous reversal, as though the early perceptual processing units are not able to handle the sensory information in the first feed-forward loop of sensory information flow (e.g., Kornmeier et al., 2011). The exogenously induced reversal (realized by the computer program) is void of this problem, which may explain the absence of the Reversal Positivity in this case. Furthermore, the subsequent three ERP components related to endogenous reversals were delayed by about 40–60 ms compared to those from the exogenously induced reversals. A comparable processing delay was observed in the reaction time data in a separate experiment and may reflect the necessary time to solve the sensory ambiguity problem.

Since its introduction, the Onset Paradigm has been used by several labs around the world and the findings, reported above, have mainly been confirmed (Kornmeier and Bach, 2004, 2006, 2012; Kornmeier et al., 2007, 2011, 2019; Pitts et al., 2007, 2008, 2010; Atmanspacher et al., 2008; Britz et al., 2010; Intaite et al., 2010, 2013; Ehm et al., 2011; Sandberg et al., 2014; Yokota et al., 2014; Abdallah and Brooks, 2020). For reviews see Pitts and Britz (2011) and Kornmeier and Bach (2012).

**FIGURE 1:**
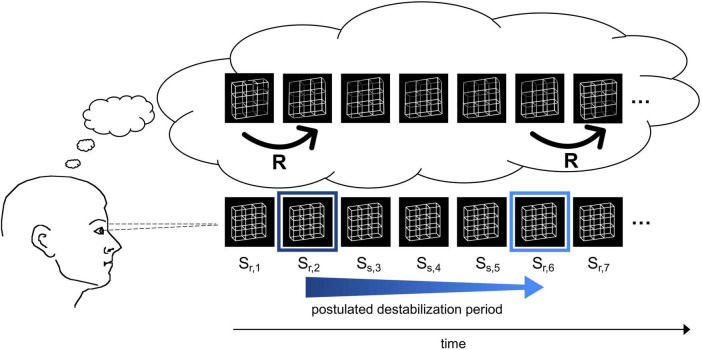
Schematic demonstration of the reversal dynamics. The participant observes an ambiguous Necker lattice discontinuously over a certain time interval and perceives it as facing in one orientation. The perceptual representation is postulated to destabilize over time (blue arrow) until the percept becomes so unstable (indicated by the light blue frame) that a perceptual reversal occurs. After the reversal, the perceptual representation is again temporally stable (dark blue frame). **Bottom row**: Necker lattice stimuli as ambiguous sensory input. **Top row**: perceptual interpretation of the observer. The instances R indicate perceptual reversals.

For the interpretation of their ERP findings, Kornmeier et al. postulated two separate processes (see Figure 1 for a schematic graphical representation): After a perceptual interpretation has been established, its neural representation starts to slowly destabilize until a state of maximal neural instability has been reached. Assuming that the brain has evolved to keep unstable states as short as possible, this instability becomes resolved by a fast restabilization process of 40—60 ms, as indicated by the ERP results, leading to the reversed perceptual interpretation (Kornmeier and Bach, 2006).

These previous EEG studies indicated that this restabilization is mainly executed in lower visual areas. However, it is so far unclear, which brain areas are affected during the postulated longer-lasting destabilization process and at which time points between two perceptual reversals we can find EEG correlates of a destabilized perceptual state. Hence, the aim of the present study is to identify EEG signatures that reflect states of gradual destabilization preceding a reversal event. Such signatures would allow us to better understand the processes underlying perceptual/neural destabilization. We focused on ERP differences between stable and unstable perceptual states in a time window before a reversal.

## 2. Materials and methods

### 2.1. Participants

EEG data from 21 participants (mean age = 25.0 years, standard deviation (SD) = 3.27 years; 11 female) was collected and analyzed. All participants had normal or corrected-to-normal visual acuity as measured with the Freiburg Visual Acuity and Contrast Test (FrACT, Bach, 2018) and gave their informed written consent. The study was approved by the ethics committee of the University of Freiburg, Germany.

### 2.2. Stimuli

**FIGURE 2:**
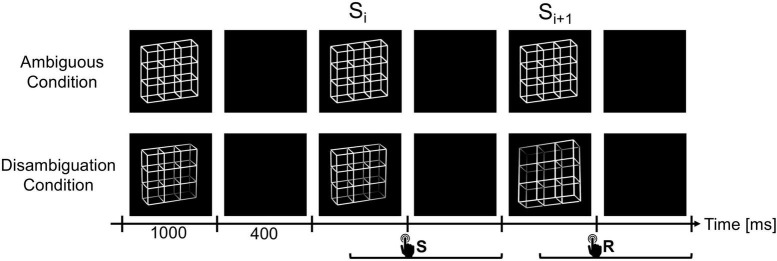
The experiment consisted of two experimental conditions, the Ambiguity Condition **(top row)** and Disambiguation Condition **(bottom row)**. In both conditions, the lattice stimuli were presented for 1000 ms with ISIs of 400 ms in between. Participants had to compare successive stimuli and indicate via button press whether they perceived the current lattice orientation as reversed compared to the previous lattice (reversal trials, R) or as unchanged (stability trials, S). Participants were allowed to respond from stimulus onset until the end of the subsequent ISI. We focused our analysis on the stimulus time window before (S_i_) and after (S_i+1_) the reversed percept, including the ISI in between. Diagram adapted from Kornmeier and Bach (2012) and Joos et al. (2020).

As visual stimuli we used the ambiguous Necker lattice (a combination of nine Necker cubes, Necker, 1832) as first described in Kornmeier et al. (2001) and Kornmeier and Bach (2004) (Figure 2) and two disambiguated lattice variants corresponding to the two perceptual interpretations of the ambiguous lattice, including depth cues, like shading, central projection, and aerial perspective (Figure 2). The lattices were white (40 cd/m^2^) with a dark background (0.01 cd/m^2^). This was the luminance for both lattice variants, the disambiguated variant’s luminance was calculated by averaging the luminance across the four corners. The Necker lattices had a size of 7.5^°^ × 7.5° degrees of visual angle. A fixation cross was presented in the center of the lattices (Joos et al., 2020).

### 2.3. Procedure

In two experimental conditions, either ambiguous or disambiguated variants of the Necker lattice were presented discontinuously (1,000 ms presentation time) with dark screen ISIs (400 ms duration and same color as the background of the lattices) between stimulus presentations. In a 1-back task, participants compared the perceived 3D orientation of the currently presented lattice stimulus (S_i+1_) with the previously presented stimulus (S_i_) and indicated perceptual reversals and perceptual stability (same percept across the two stimulus presentations) by different keys. We kept the stimulus presentation time short enough (1,000 ms) to prevent additional reversals during stimulus observation. In the case of ambiguous lattice stimuli, perceptual reversals across stimuli were evoked endogenously by the participants’ perceptual system. In the case of the disambiguated lattice variants, the reversal was evoked by the computer program with a predefined reversal rate of 30%, reflecting average reversal rates of a Necker cube stimulus (Kornmeier et al., 2009). The two experimental conditions were subdivided in experimental blocks of approximately 7 min duration, containing typically some 300 trials.

Before the experiment, participants completed a training session where they were presented with disambiguated Necker lattices. Identical to the experiment, participants needed to indicate with a button press if the percept stayed the same or changed from S_i_ to S_i+1_. The training duration was approximately 5 min. If participants got less than 90% of the trials correct, the training was repeated.

### 2.4. EEG recording

The EEG was recorded with 32 active silver/silver chloride electrodes using the extended 10–20 system for the electrode positions on the scalp (American Clinical Neurophysiology Society, 2006), using the BrainVision ActiCHamp amplifier. The data was digitized at a sampling rate of 1000 Hz and online bandpass filtered at 0.01–120 Hz. Impedance was kept below 10 kΩ for all electrodes.

### 2.5. EEG pre-processing

All pre-processing was done using the MNE-Python package (version 0.23.0) (Gramfort et al., 2013) in Python 3.9.6. The raw data was offline band-pass filtered from 0.01 to 25 Hz and re-referenced to the average of the mastoid electrodes (TP9, TP10). The vertical electrooculogram (vEOG) electrodes were used to determine eye blink artifacts. Due to technical problems, one participant did not have a vEOG electrode, and therefore the Fp1 electrode was used for artifact detection. The vEOG electrode (or Fp1 electrode) was bandpass filtered from 1 to 10 Hz.

To detect eye blink artifacts an Independent Component Analysis (ICA) was conducted. A correlation was calculated between the vEOG electrode and the Independent Components (IC) to detect which components were most correlated to eye movements. If a component surpassed a predefined correlation threshold of *r* = 0.8, this component was eliminated from the data.

For the remaining artifacts an artifact rejection threshold of ±100 μV (peak-to-peak amplitude) was defined. For the Ambiguous Condition, this resulted in an average (across participants) of 24% of trials being dropped, with a standard deviation of 21%. For the Disambiguation Condition 22% of trials were dropped, with a standard deviation of 20%. Data was baseline corrected, with the average amplitude in a time window between 60 ms before and 40 ms after stimulus onset of S_i+1_. Trials were labeled as either reversal or stable depending on the participants’ responses to stimulus S_i+1_. Responses were regarded as valid if given in a time from 150 ms after S_i+1_ onset to the end of the ISI following S_i+1_. If a response button was pressed more than once during this response time interval, the latest response was used. In cases of pressing wrong buttons or no responses the trial was ignored.

### 2.6. Data analysis

For the data analysis we collected reversal trials S_r,i+1_, where the perceptual reversal took place from stimulus S_r,i_ to stimulus S_r,i+1._ We compared these reversal trials with stability trials S_s,i+1_, where perception stayed unchanged from S_s,i_ to S_s,i+1_. Our specific focus was whether the EEG from the trials S_r,i_ and the subsequent ISI preceding reversal trials S_r,i+1_ differed from the stability trials S_s,i_ and the subsequent ISI.

#### 2.6.1. Reversal rates

We calculated the number of reversals per minute (reversal rate) by counting the number of reversals per experimental block and dividing the sum by the block duration. One block lasted for 7 min on average. This reversal rate was then averaged within participants across experimental blocks of the same condition. The focus of the present analysis was the identification of EEG signatures related to perceptual instability before a reversal compared to a time window of perceptual stability. For this purpose, it was necessary to have a minimum number of reversal trials per participant. We therefore excluded participants with less than 5 reversals per minute, resulting in a dataset that included 15 participants from the original 21. Table 1 shows the average reversal rates per minute for every participant.

**TABLE 1:** Number of average reversals per minute for every participant.

Participant	Average reversals per minute
**A**	**8.2**
**B**	**16.75**
**C**	**6.5**
**D**	**16.2**
**E**	**11.6**
**F**	**8.22**
**G**	**10.33**
H	3.44
**I**	**11.78**
**J**	**10.67**
K	4.33
L	1.22
M	0
**N**	**18.33**
**O**	**12.11**
P	4.56
**Q**	**8.89**
**R**	**17.22**
**S**	**16.44**
**T**	**9.33**
U	0.22

The bold participants are the ones used in the analysis.

#### 2.6.2. Global field power (GFP)

For each participant, we calculated event-related potentials by separately averaging S_r,i_ and S_r,i+1_ across reversal trials and S_s,i_ and S_s,i+1_ across stability trials. We then used the ERPs to calculate the Global Field Power, “GFP,” which is the spatial standard deviation at a single time point (t) (Lehmann and Skrandies, 1980).


$$G\,F\,P\,\left(t\right)=\sqrt{\frac{\sum_{j=1}^{n}{\left(v\,{\left(t\right)}_{j}-\bar{v}\,\left(t\right)\right)}^{2}}{n}}$$

where *v*(*t*)_*j*_ is the voltage at electrode *j* at time point *t*, $\bar{v}$ is the mean voltage of all electrodes at time point *t*, and *n* is the total number of electrodes. The GFP is calculated for every time point, resulting in a time series of GFP values.

We were interested in EEG correlates of upcoming perceptual reversals. Given, that it is so far unclear at which time point or at which EEG electrode exactly we should expect such correlates, we used the GFP as a measure that integrates across electrodes and thus reduces the dimensionality of the data. Results from the GFP then allowed us to define a temporal region of interest (ROI) for the next analysis steps.

#### 2.6.3. Data analysis based on machine learning

To investigate how much the EEG data differed between stable and reversal trials, an Artificial Neural Network (ANN) was trained. For this we used the Deep4Net ANN via the package Braindecode, version 0.5.1 (Schirrmeister et al., 2017), which was specifically designed for the analysis of EEG data. The data the network received was each participant’s single trials and all the electrodes. The ANN was trained and evaluated individually for every participant in the following way:

(a) From each participant’s trials, 75% were systematically selected for the training and testing of the ANN and the remaining 25% were selected for the evaluation of the ANN. For the training procedure the order of trials was randomized and the reversal trials resampled to match the number of stable trials (the evaluation trials were not randomized or resampled). The data were also all normalized, resulting in all values in a single trial to range between 0 and 1.
As a result of the evaluation, for each trial from the evaluation set we either obtained a 1 or 0 as a “*prediction label*” from the ANN classification, indicating either the identification of a stable or an upcoming reversal trial. Additionally, for each trial we obtained a binary manual response from the participant indicating either a perceived reversal or perceived stability, which we call “*true label.*” We then calculated *accuracy values* as the number of evaluation trials where the predicted label matched the true label, divided by the total number of trials in the evaluation set.
(b) This procedure was executed four times to have each quarter once for the evaluation, resulting in four separate accuracy values in percent discriminability. For each participant, we calculated the median of the resulting four accuracy values. The median accuracy reflects the differentiation between stable and upcoming reversal trials, ideally, indicating how good the destabilization of a perceptual state is reflected in the EEG data. Additionally, and more realistically, the accuracy also reflects how good the ANN was at extracting the relevant information.

One basic idea of this study was, that in the case of endogenous spontaneous perceptual reversals, during observation of an unchanged ambiguous cube stimulus, the EEG contrast described above may reflect a state of perceptual destabilization immediately before the reversal. In contrast, in the case of exogenously induced reversals between the disambiguated cube variants, no such destabilization state should be observed. This should be reflected in a comparison between the ambiguous and disambiguated stimulus conditions.

We applied a bootstrap method to compare distributions of accuracy values for the two conditions in the following way:

(c) We put all the test results from all four test sets together, randomly selected 70% of these data and calculated an accuracy value from these data. This was done separately for each participant and experimental condition (ambiguous and disambiguated stimuli) 1,000 times in a sampling-with-replacement manner.
(d) For each participant we created two accuracy value distributions for the two conditions and compared them with each other by applying a Kolmogorov-Smirnov test.

The analysis steps (a)—(d) were applied for two different temporal ROIs, which were (1) a time window between 300 and 700 ms after onset of the stimulus immediately before an indicated perceptual reversal (S_i_) and (2) the subsequent ISI. The choice of the S_i_ time window was based on the results from the previous Global Field Power analysis. Within the time range where the *p*-values comparing the reversal and stable GFP in S_i_ were below 0.05, a 400 ms time window was chosen to match the time window length of the ISI. The beginning of the time range was determined by finding the first time point where the *p*-values were less than 0.05 after onset of S_i_ and physiologically plausible [150 ms after stimulus onset (Joos et al., 2020)]. This was 304 ms, and therefore, it was rounded down to 300 ms.

#### 2.6.4. Estimating spatial regions of interest

The EEG is a rough measure with a relatively low signal-to-noise ratio. Whether a brain signal of interest can be measured on the scalp, depends on a number of factors, like individual brain anatomy, conductivity and thickness of bones and meninges, etc. (e.g., Bach, 1998; Nunez and Srinivasan, 2006). As a consequence, such a signal may be clearly identified in some participants, but be apparently absent in others, even if the neural processes of interest may be highly similar across participants. For this reason, we performed the above-described analysis on the level of individual participants and selected indicative participants (as defined below) to investigate, which of the 32 EEG electrodes provided the most classification information. We identified 8 indicative participants (see section “3. Results”).

The ANN then was trained and evaluated 8 more times on the indicative participants and with each training and evaluation a subgroup of electrodes (frontal, central, parietal, occipital, left hemisphere, right hemisphere, parietal left hemispheric, and parietal right hemispheric electrodes) was removed. If the accuracy was lower when removing some electrodes compared to others, this suggested that the missing electrodes contained useful information for the ANN to classify the EEG of S_i_ as a reversal or stable trial.

To quantify the results of these different ANN runs, a ratio between the accuracy values from the Ambiguity Condition and Disambiguation Condition was calculated for each participant. The Disambiguation Condition in this case served as a control as the EEG from the trial immediately before the exogenously induced reversal (S_i_) should have no information about the subsequent reversal (S_i+1_). As a consequence, no indication of perceptual reversals should be observed in the data from this condition.

#### 2.6.5. Source analysis

Standard EEG electrode positions were assumed for all *q* = 32 electrodes according to the 10–20 system since no individual EEG electrode positions from the individual participants were available from the dataset. We used the “fsavagerage” (Fischl et al., 1999) template T1 image as provided by the Freesurfer image analysis suite^1^. The forward model was computed using the boundary element method (BEM, Fuchs et al., 2002) as provided by MNE-Python (Gramfort et al., 2013). Each shell (brain, skull, and scalp tissue) was composed of 5120 vertices. Conductivity was set to 0.3 S/m for brain and scalp tissue, and 0.06 S/m for the skull.

The source space was created using *p* = 1284 dipoles with icosahedral spacing, i.e., dipoles were placed along the cortical folding by iterative subdivision of a icosahedron (cf.^2^, Gramfort et al., 2013). In order to reduce computational complexity and based on the reasonable physiological assumption, a fixed orientation of dipoles orthogonal to the surface of the cortical sheet was assumed.

Inverse solutions were calculated for the ERPs spanning 300–700 ms after the onset of S_i_. This was the same range used for the classification of the ANN as described above. In order to mitigate the problem of missing individual forward models, we adapted a group-inversion scheme similar to the one described by Friston et al. (2015).

As a first step, we concatenated the reversal- and stability-ERPs of all participants. Next, we identified a global set of active sources by calculating the flexible multi-signal classification (FLEX-MUSIC) inverse solution on the concatenated ERPs (Hecker et al., 2023). FLEX-MUSIC is a recently developed improvement of the well-established recursive MUSIC approach to solve EEG inverse problems, that can accurately estimate not only the location but also the spatial extent of neural sources. Finally, weighted minimum norm estimates [wMNE, (Pascual-Marqui, 1999)] were calculated on the global set of active sources for each participant and condition.

## 3. Results

### 3.1. Global field power already shows differences in conditions approximately 1,100 ms before the reversal

**FIGURE 3:**
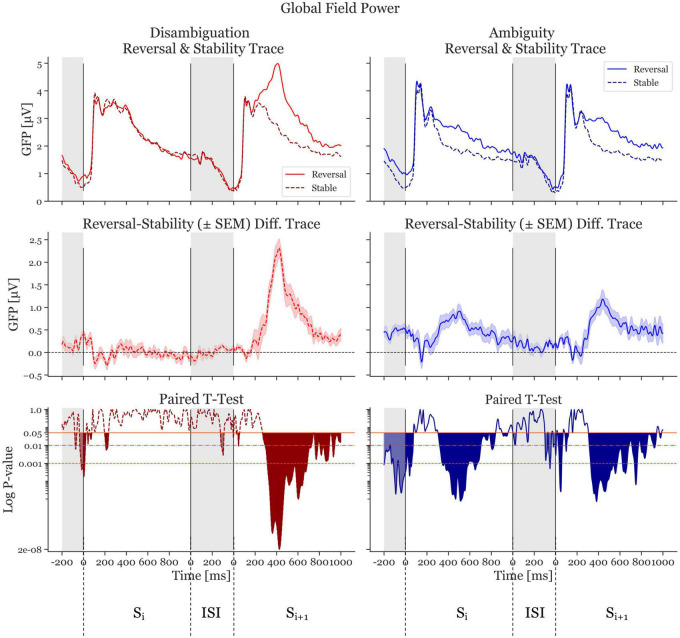
Global Field Power (GFP) and *p*-values of the Disambiguation (red) and Ambiguous (blue) Conditions. The two **top panels** show the GFP averaged across participants. The dashed, darker traces depict the stable condition, whereas the lighter, continuous trace depicts the reversal condition. The **middle two panels** depict difference GFP traces of the top row (reversal minus stability) resulting in the difference traces. The shaded area is ± standard error of the mean. The **bottom panels** show the *p*-values logarithmically scaled. The orange-red horizontal lines depict alpha values of 0.05, 0.01, and 0.001, respectively. The filled (red and blue) areas indicate statistically significant time periods. The gray areas indicate interstimulus interval time ranges. The first range shown is the ISI preceding S_i_. The next 1,000 ms represent the onset period of S_i_ and the 1000 ms after the interstimulus interval show S_i+1_.

The GFP of reversal- and stable-ERPs, together with the GFP difference traces (reversal GFP minus stability GFP) are shown in Figure 3. From this Figure, it is clear that in the Disambiguation Condition (red traces, left), the activity in S_r,i_ is similar to that of S_s,i_. As a consequence, the difference GFP trace in the S_i_ time window is close to zero. The GFP difference trace in the S_i+1_ time window, however, shows a highly significant deviation from the zero line, indicating a clear GFP difference between a reversed and a stable percept (maximal significance at 427 ms after onset if S_i+1_, *p* = 1.6 × 10^–8^, Cohen’s *d* = 2.19).

The Ambiguity Condition (Figure 3, blue traces, right) presents a different pattern. The difference GFP trace in S_i_ shows a significant deviation from zero, with a similar shape to the trace from S_i+1._ The most significant effect in the S_i_ time window is at 514 ms after stimulus onset (*p* = 7.59 × 10^–6^, Cohen’s *d* = 1.35). The most significant effect in the S_i+1_ time window is at 382 ms after stimulus onset (*p* = 7.3 × 10^–6^, Cohen’s *d* = 1.06).

### 3.2. ANN uses parietal information to classify reversal and stable trials already approximately 1,100 ms before an upcoming reversal

**FIGURE 4:**
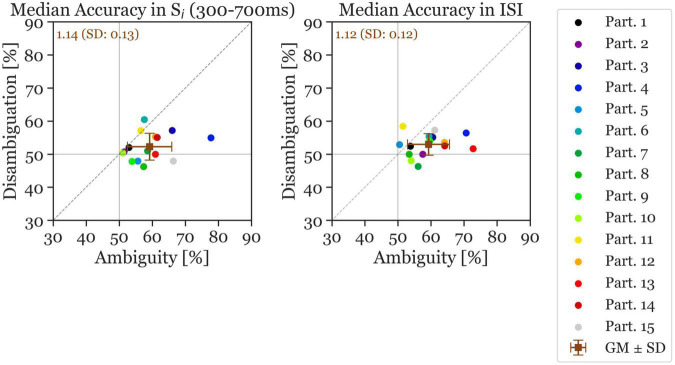
Median accuracy of individual participants (colored circles) and the grand mean ± standard deviation (brown squares). The x-axis shows the accuracy in the Ambiguous Condition and the y-axis the accuracy in the Disambiguation Condition. The **left panel** shows the accuracy calculated during the S_i_ stimulus time window and the **right panel** shows the accuracy during the ISI time window (reversal and stable). The number inserted in the top left shows the mean accuracy ratio (ambiguous divided by disambiguated) accuracies (with the standard deviation). The continuous gray horizontal and vertical lines indicate chance level at 50% accuracy.

First, the ANN was trained and evaluated on the most statistically significant 400 ms of the stimulus presentation window S_i_ based on the GFP results (i.e., 300–700 ms after stimulus onset, cf. Figure 3). This resulted in a mean accuracy of 59.22% for the Ambiguity Condition and 52.29% for the Disambiguation Condition (with 50% being chance level). Cohen’s d was 1.21 comparing the Ambiguous and Disambiguated Accuracies for S_i_. Figure 4 indicates that for all but two participants the ANN results showed a higher accuracy in the Ambiguous Condition compared to the Disambiguation Condition.

Next, the ANN was trained and evaluated on the 400 ms ISI, resulting in a mean accuracy across all participants of 59.26% for Ambiguous and 53% for Disambiguated Conditions (Cohen’s d = 1.21). Again, all but the same two participants as mentioned previously had a higher accuracy in the Ambiguous Condition during the ISI. The average accuracy ratio between ambiguous and disambiguated is slightly larger in S_i_ (1.14) compared to during the ISI (1.12). The standard deviation of the accuracy ratios is also larger in S_i_ (0.13) compared to during the ISI (0.12). Moreover, the accuracies show more variance in the Ambiguity Condition than in the Disambiguation Condition.

**FIGURE 5:**
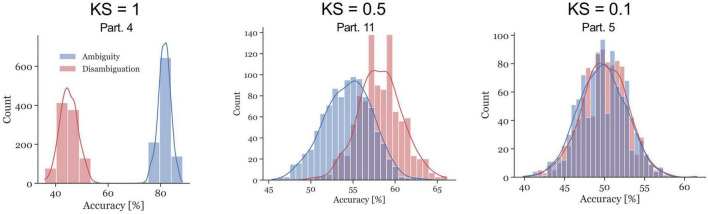
Examples of accuracy distributions resulting from the bootstrap method and the corresponding Kolmogorov–Smirnov (KS) test statistic for participants 4, 11, and 5. The red distributions represent the Disambiguation Condition accuracies, the blue distributions represent the accuracies in the Ambiguous Condition. The KS test statistics ranges between 0 and 1. The closer the values are to 1, the further apart the distributions are.

**FIGURE 6:**
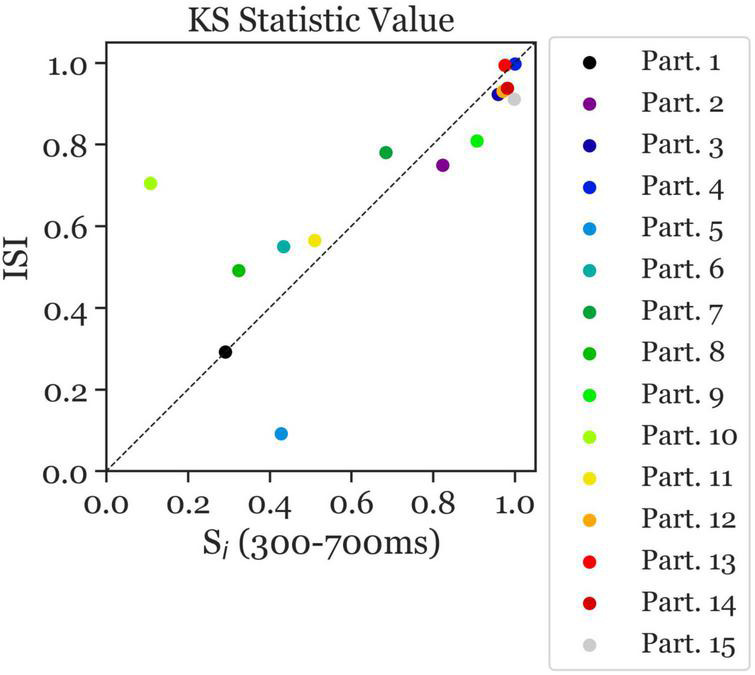
Kolmogorov–Smirnov (KS) test statistic values indicating the separability of the distribution of ambiguous and disambiguated accuracies in S_i_ (300–700 ms) and the ISI. The KS test statistic values ranges from 0 to 1. The closer the values are to 1, the further apart the distributions are. The x-axis presents the KS statistic of S_i_ Time Window. The y-axis presents the statistic of the ISI Time Window. The colors correspond to the individual participants. Most participants with high/low separability in the S_i_ Time Window also showed high/low values in the ISI Time Window, with overall better separability in the S_i_ Time Window than in the ISI Time Window (i.e., more data points below the diagonal). Moreover, largest separability can be observed in participants 2, 3, 4, 9, 12, 13, 14, and 15 (indicative participants).

The ambiguous and disambiguation accuracy distributions were compared, in both the 300–700 ms in S_i_ and the 400 ms of the ISI, using the Kolmogorov-Smirnov test. The Kolmogorov–Smirnov test was used to quantify the difference between the accuracy distributions of the Ambiguous and Disambiguated condition for each participant. Figure 5 displays distributions based on bootstrapping from three representative example participants together with the related test results. The three examples indicate the strong differences in the discriminatory power of the EEG between participants. The Kolmogorov-Smirnov test results from all participants is shown in Figure 6. One interesting aspect in this graph is the linear relation between the ISI and S_i_ data, indicating that if a participant’s EEG showed a strong discriminatory power in the S_i_ time window, it was also relatively strong in the subsequent ISI. The correlation between the statistic values of these two time windows was ρ = 0.8 (*p* = 0.0009). Furthermore, 60 % of participants showed larger discriminatory power in the S_i_ time window compared to the ISI time window. Finally, the graph identifies participants 2, 3, 4, 9, 12, 13, 14, and 15 as indicative participants. Participant 13 has more discriminatory EEG power in the ISI compared to S_i_, while for the others there is more discriminatory power in S_i_ compared to the ISI. The indicative participants were determined by choosing all participants that had a KS statistic score larger than or equal to 0.8 in either the S_i_ time window or the ISI time window.

In the following spatial analysis steps, we focused on these 8 indicative participants. We repeated the ANN calculations with different subsets of electrodes in order to identify electrode subsets that were necessary to obtain high discrimination performance.

**FIGURE 7:**
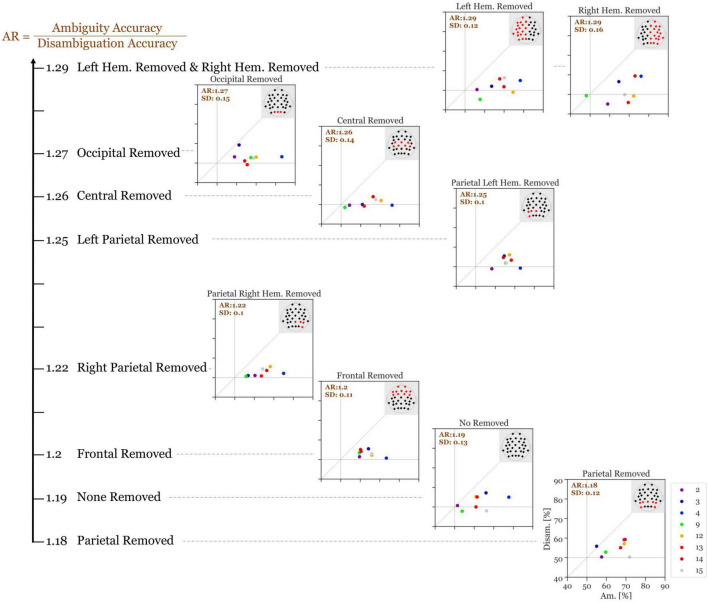
Accuracy ratio of S_i_ (300–700 ms after stimulus onset) of the 8 indicative participants with different electrode subsets removed with each run. The values show the mean accuracy ratio of all indicative participants. The different colors depict the different participants and the numbers in the top left of each panel represents the mean accuracy ratio (Ambiguity divided by Disambiguation; with the standard deviation). The larger the distances (to the right and to the top) of the individual icons from the horizontal and vertical gray lines are, the higher is the discriminatory power of the respective participant’s EEG data.

Figure 7 shows the results of this analysis. Without the removal of any electrodes, our high-performance participants had an average accuracy ratio of 1.19 (SD = 0.13). When the parietal electrodes were removed, the median accuracy was slightly decreased (accuracy ratio = 1.18; SD = 0.12). Remarkably, for all other variants of electrode removal, the accuracy increased. Moreover, removing the left parietal electrodes increases the accuracy slightly more than removing the right parietal electrodes (accuracy ratio = 1.25; SD = 0.095 versus accuracy ratio = 1.22; SD = 0.098). Additionally, removing all electrodes from the left hemisphere increased the accuracy slightly more than removing the right hemisphere electrodes (accuracy ratio = 1.30; SD = 0.12 vs. accuracy ratio = 1.29; SD = 0.16). Overall, removing the left-hemisphere electrodes provides the highest accuracy ratio among all variants.

### 3.3. Source localization

Source localization was performed in a two-stage procedure. First, the global set of active sources was identified with the EEG source analysis method FLEX-MUSIC, then the neural activity at these regions was estimated on the level of individual participants.

**FIGURE 8:**
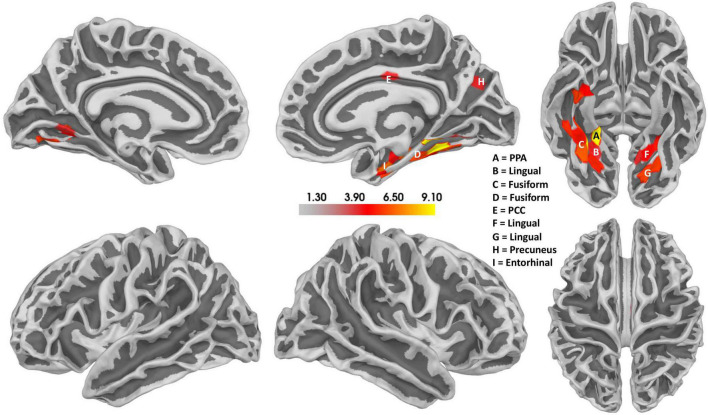
Results of the source localization with thresholded *t*-values for the Ambiguity Condition. Reversal ERPs compared to stable ERPs. The size of the cluster does not represent the relevance of the cluster. Activity seems to be concentrated mainly in the right hemisphere, specifically the parahippocampal place area (PPA). There also seems to be some activity in the left hemisphere in a similar area. Additionally, the posterior cingulate cortex (PCC) also seems to be active.

Notably, a relatively confined set of active regions was found (cf. Figure 8), which is remarkable given the presumably high inter-individual variance of the EEG data. The results of the source localization suggest that the parahippocampal place area (PPA) most significantly differs between reversal and stable trials (*p* = 3.96 × 10^–05^; cf. Figure 8 and Table 2). A summary of the brain areas and the corresponding *t*- and *p*-values is shown in Table 2. Other regions that significantly differ are the fusiform gyrus, the lingual gyrus, the entorhinal cortex, the posterior cingulate cortex, and the precuneus. Most activity seems to be located in the right hemisphere.

**TABLE 2:** The location of all significant voxels that are in named brain areas in order of most significant to least significant.

Brain area	Hemisphere	Legend in Figure 8	T	p
Parahippocampal place area	Right	A	9.1	3.96 × 10^–5^
Fusiform gyrus	Right	C	9.1	4.09 × 10^–5^
Lingual gyrus	Left	G	6.34	0.0004
Fusiform gyrus	Right	C	6.12	0.0005
Lingual gyrus	Left	F	5.74	0.0007
Fusiform gyrus	Right	D	5.71	0.0007
Fusiform gyrus	Right	D	5.68	0.0008
Lingual gyrus	Left	G	5.64	0.0008
Fusiform gyrus	Right	C	5.37	0.001
Lingual gyrus	Right	B	5.22	0.001
Lingual gyrus	Left	F	5.98	0.002
Fusiform gyrus	Right	B	4.93	0.002
Entorhinal cortex	Right	I	4.67	0.002
Lingual gyrus	Left	F	4.65	0.002
Posterior cingulate cortex	Right	E	3.82	0.007
Lingual gyrus	Right	B	3.81	0.007
Lingual gyrus	Left	F	3.79	0.007
Precuneus	Right	H	3.73	0.007

## 4. Discussion

The primary aim of the present study was to identify EEG correlates of a destabilized perceptual brain state preceding a spontaneous perceptual reversal of an ambiguous Necker lattice. Given the absence of spatial and temporal regions of interest from the EEG literature to focus this question on, we started with a Global Field Power (GFP) analysis. We compared the GFP from time periods during perceptual stability with those about 1 s before a perceptual reversal and found large GFP effects with ambiguous lattice stimuli. As expected, no such effects were observed with the disambiguated control lattice stimuli.

The concept of perceptual destabilization is not well established in the literature. It is, thus, unclear, how common the underlying neural processes are in time and brain areas across participants. We, therefore, focused the analysis steps subsequent to the GFP analysis on the level of individual participants using ANNs. We determined the accuracy of the individually trained ANNs to discriminate between stable and destabilized brain states. This analysis identified eight (out of 15) participants with high EEG discrimination performance already about 1 s before an upcoming reversal. By repeated re-analysis of the data from these eight participants with the systematic removal of subsets of electrodes, we were able to identify the parietal cortex as a critical brain area for the prediction of an upcoming reversal. A subsequent source localization analysis revealed the parahippocampal place area (PPA) as the most statistically significant brain area for reversal prediction. Previous studies identified a network of frontal and parietal regions being active and relevant during spontaneous perceptual reversals (Brascamp et al., 2018; Watanabe, 2021). Interestingly, neither our ANN analysis nor the source analysis indicated the relevance of this network for destabilization. The source localization needs to be taken with caution, due to the missing individual forward models.

### 4.1. Potential limitations

#### 4.1.1. Limited number of trials and participants

In the present study, we measured 21 participants. Six had to be excluded due to too low reversal rates (see section “2. Materials and methods”), resulting in a dataset of 15 participants. This is not a very large sample to make strong claims about common mechanisms in the general population. On the other hand, the majority of our analyses was realized on the level of individual participants. From our 15 analyzed participants, we identified eight indicative participants. We cannot say whether the effects we identified in 53% of our participants can also be identified in 53% of the population. However, we regard our results as a good starting point for follow-up studies with a specific focus on perceptual representations and their stability criteria. Further below, we will also suggest possible next steps to increase the discrimination accuracies.

#### 4.1.2. Are perceptual reversals during discontinuous stimulus presentation a good model for the continuous case?

The introduction section contains an entire paragraph on the question about how to measure spontaneous endogenous perceptual reversals of an ambiguous stimulus with high temporal resolution. In the present study, we used the Onset-Paradigm as a possible way to address this principal problem. One can now ask whether a changed percept from one stimulus presentation to the next is really comparable with a spontaneous reversal during a continuous observation of the ambiguous stimulus?

We think “yes,” as we have already discussed this question in detail in our review paper in 2012 (Kornmeier and Bach, 2012). In short, continuous observation is never really continuous, because of around 15 eye blinks per minute on average (Sforza et al., 2008), with a duration of around 200 ms (Caffier et al., 2003) and the ensuing saccadic suppression (Binda and Morrone, 2018). Moreover, a number of behavioral studies showed a continuous transition of reversal rates from continuous stimulus presentation to short reoccurring interruptions up to 400 ms duration (Orbach et al., 1963, 1966; Kornmeier et al., 2007). Clearly, however, interrupted stimulus presentation is not the same as continuous presentation. Based on the present results, it may thus be an interesting next step to train ANNs with EEG data from discontinuous presentation and apply the trained ANN to EEG data from continuous observation.

#### 4.1.3. Can the findings from the Necker lattice be generalized across other types of ambiguous stimuli?

We interpret our findings as neural destabilization correlates of perceptual representations. We have already discussed that neither the concept of perceptual representations and their neural correlates nor the concept of destabilization are well established. This becomes obvious, if we compare perceptual reversals of ambiguous figures with perceptual reversals of binocular rivalry stimuli, as recently discussed by Bachmann and Aru (2023). In the case of ambiguous figures, the stimulus features, e.g., the edges of the Necker cube, stay in consciousness but are interpreted differently. As a consequence, only a part of the perceptual representation may become destabilized in the context of a reversal. In the case of binocular rivalry, only the input from one eye enters consciousness, whereas the input from the other eye stays unconscious (if we ignore the intermediate patchwork periods between two distinct percepts). In this case, the whole “perceptual representation” may become destabilized. It may thus be interesting to replicate the current experiment with binocular rivalry stimuli, to see how much of our results can be replicated.

### 4.2. Why do we only see clear discrimination accuracy in 53% of the participants?

The scalp EEG is a relatively rough measure. Whether a neural signal reaches the scalp and can be detected by the scalp EEG electrodes depends, amongst other factors, on the individual folding of the brain. The activity of neighboring brain sources of activity can be superimposed and weaken each other. Furthermore, neural signals related to a certain processing step are typically superimposed on background activity, not necessarily related to this processing, but affecting the signal-to-noise ratio of the signal in question (Arieli et al., 1996; Bach, 1998). These and other factors contribute to a large inter-individual variability of brain activity, even if participants observe the same stimulus or execute the same task (e.g., Kornmeier et al., 2014). An impressive demonstration of the inter-individual variability during observation of multistable stimuli has recently been presented by Wexler (2018). A huge number of reported EEG effects, including work from our lab, resulted from group statistics and were difficult to observe in a large number of individual participants (e.g., Ehm et al., 2011). Hence, it is not surprising that in the present data some participants showed weak or even no effects. The low signal-to-noise ratio of single-trial EEG may also explain that even for the eight indicative participants the discrimination accuracy remained below 80%. Finally, another potential limitation could be the low number of trials the ANN had available for training. Due to potential fatigue effects in our participants, typically resulting in rising numbers of body movement artifacts, wrong key presses etc., it was difficult to increase the number of trials to be enough for training the ANN.

Beyond these rather technical reasons, another interesting more functional factor may play a role. Polgári et al. (2020) recently published an interesting study about eye-movements during observation of the Necker cube. In the present study, participants were instructed to fixate on a fixation cross in the center of the cube stimuli in order to minimize eye-movement artifacts in the EEG. In Polgári et al.’s (2020) study participants were allowed to freely move their eyes. The authors analyzed fixations and clustered them based on the horizontal fixation location as an either right or left fixation cluster with respect to the vertical midline of the visual field in which the Necker cube was presented centrally. They found that in most cases, in which participants reported a reversal, the eyes also moved from one cluster to the other. However, they recorded about twice as many changes of the horizontal eye position between clusters than reversals, which means that not every cluster change was accompanied by a clear and conscious perceptual reversal. Polgári et al.’s (2020) explanation of this observation is in contrast to the basic assumption underlying the present study. We *a priori* assumed that participants’ percepts become stable immediately after a reversal and only slowly destabilized toward the next perceptual reversal. Comparing the EEG from the middle of this proposed temporal window of perceptual stability with the EEG close to a reversal should therefore reveal potential EEG correlates of perceptual stability state differences (stable versus destabilized). Polgári et al. (2020) in contrast, postulated that the perceptual brain state can also destabilize in between two button presses, but sometimes restabilizes back to the perceptual interpretation where it started. They further speculate that such unconscious destabilizations may come with a partial or total departure from one of the clustered horizontal eye positions, which would explain the larger number of cluster changes than button presses. Interestingly, indications of unconscious destabilizations during observation of ambiguous figures have also been reported by other groups (e.g., Pastukhov and Braun, 2007; Pastukhov and Klanke, 2016).

As described above, our discrimination accuracy measure is based on a postulated EEG contrast between perceptually stable and unstable brain states. If Polgári et al.’s (2020) postulate of unconscious destabilizations is correct, it may be possible that the time windows we selected and labeled as perceptually stable brain states between two button presses may contain periods of unconscious destabilizations. This may lead to a larger variability and a smaller signal-to-noise ratio of our measure. Accepting Polgári et al.’s (2020) interpretation, horizontal eye-movement data can be used in a future replication of the present study to better determine perceptually stable brain states.

### 4.3. What can the current results contribute to the understanding of perceptual representations?

When our participants look at a black screen and suddenly a white lattice stimulus occurs, a clear and distinct pattern of EEG activity can be identified within the first 600—800 ms after stimulus onset. This pattern typically has a temporal profile which can be observed, e.g., in the ERP with stimulus onset as the reference time point. In the first 300 ms we see so-called exogenous signatures with typically sharp and relatively large deviations from baseline, followed by broader components, like the P300-family (e.g., Figure 2C in Kornmeier and Bach, 2006). These signatures in the ERP (lower-frequency range) are typically accompanied by modulations in higher frequencies (Ehm et al., 2011). These signatures are typically discussed as processing steps along a hierarchy of perceptual processing on the way to create a stable perceptual interpretation (VanRullen and Thorpe, 2002; Dehaene and Changeux, 2011; Kornmeier and Bach, 2012; Sergent et al., 2017). Interestingly, after the percept has been established and is stable, the measurable signals seem to fade out, raising the fundamental question of how stable perceptual interpretations are represented in the brain over time (e.g., Krüger, 2022). Of course, given the quality of the EEG, we cannot infer that the absence of a measurable signal means no processing in the brain. At this point, ANNs become relevant, because these methods seem to be more sensitive to subtle signals.

Given that no precise spatio-temporal regions of interest were available for our experimental question and taking into account, that the signatures we were looking for, could be highly variable in space and time across observers and at the same time very small, we decided to focus on within-participant statistics and apply ANN methods for our analysis after a group GFP analysis. In the GFP analysis of the Ambiguity Condition we found surprisingly large effects in a time window already about 1 s before a reversed percept was established. Two observations are of particular interest here:

1. In our analysis, we focused on two time windows of interest, the S_i_ time window, where the last lattice stimulus before the perceptual reversal was presented, and the subsequent ISI. In the GFP analysis we found a relatively long time period (about 400 ms) of significance with huge effect sizes up to 1.35. We also found significant effects in the subsequent ISI, but with much shorter time periods and smaller effect sizes.
2. Interestingly, while the GFP analysis indicates that most of the destabilization information seems to necessitate the presence of a stimulus (S_i_ time window), the subsequent ANN analysis draws a more sophisticated picture. In contrast to the GFP results, Figure 6 indicates that our indicative participants showed comparable discriminatory power in both the S_i_ time window and in the subsequent ISI. This supports the observation that the ANN methods are more sensitive. Moreover, it indicates that the destabilization state of the perceptual system can also be read out from EEG data in the absence of a visual stimulus.

During the S_i_ presentation time window, the period of high significance started at about 300 ms after stimulus onset. Interestingly, 300 ms are discussed as an estimate of time, necessary for a perceptual interpretation to become conscious, after the early visual processing steps have finished (Atmanspacher et al., 2008; Dehaene and Changeux, 2011; Kornmeier and Bach, 2012; Atmanspacher and Filk, 2013; Kornmeier et al., 2017a). If we interpret our observed effects as correlates of perceptual destabilization, then the GFP analysis results indicate, that the early visual processing units are less relevant. This hypothesis is further supported by the observation that eliminating the occipital electrodes did not substantially reduce the discriminatory power (cf. Figure 7).

Our current results do not allow strong conclusion about the “nature of a perceptual representation,” nor on the definition of precise spatial regions of interest for subsequent studies. However, the huge effect sizes in precisely defined time windows make this study a perfect starting point for subsequent studies about stable and unstable perceptual brain states.

## 5. Conclusion

We (Ehm et al., 2011) and others (Britz et al., 2009, 2010; Nakatani and van Leeuwen, 2013) already reported about EEG effects shortly before a reversed percept of an ambiguous Necker lattice is established. Our present results indicate that in some participants such anticipatory activity can be identified already at least 1 s before the reversal. Technical reasons (not enough single trials and number of reversals), unfortunately, did not allow us to go further back in time. It is, thus, possible that EEG indicators of destabilization and, thereby, of an upcoming perceptual reversal are present earlier. It would be highly interesting to see whether this is really the case. Moreover, in the present study, this anticipatory activity was only found in a subgroup of our participants. It is currently unclear, whether similar processing is also present in the other participants but is not measurable with EEG. Follow-up studies with more trials and thus a better signal-to-noise ratio may be able to answer this question.

In his Principles of Psychology, the great psychologist William James wrote about stable mental representations and transient, unstable states between them (James, 1890). He emphasized that the stable states are the “substantive parts” that enter consciousness, while the “transitive parts” are typically very fast and stay unconscious. The basic idea of this has further been elaborated in terms of categorical versus a categorical perceptual/mental states (Atmanspacher, 1992; Feil and Atmanspacher, 2010; Atmanspacher and Fach, 2019).

The present findings indicate that the perceptual system is already in an unstable state about 1 s before a perceptual reversal. The eye-tracking data from Polgári et al. (2020) further indicate that transient periods of perceptual instability may occur between two consciously perceived reversal events. All of this indicates that what James described as the transient state may only be the tip of the iceberg, i.e., the point of maximal instability (Kornmeier and Bach, 2012) and that perceptual instability is a gradual and longer lasting phenomenon rather than being binary and short.

We demonstrated that perceptual instability can be measured with the EEG. Moreover, the effects are large enough to be visible in a considerable number of individual participants. Taking into account the findings from Polgári et al. (2020) it may even be possible to increase the sensitivity of our measures by eliminating time windows of apparent stability, in which eye tracking data, however, would indicate destabilization. This might make some of the non-indicative participants become indicative participants.

We interpret the present EEG effects as a measure of perceptual instability, i.e., as reflecting the difference in neural activity between stable and destabilized perceptual states. An interesting question for subsequent studies, is to see whether this measure reflects perceptual (in)stability on a continuous scale, i.e., whether the size of the effect reflects the amount of instability of the system. Furthermore, a number of studies show deviating perceptual reversal dynamics, when participants with psychiatric disorders observe ambiguous stimuli (e.g., Notredame et al., 2014; Schmack et al., 2015; Robertson et al., 2016; Kornmeier et al., 2017b). These deviating perceptual dynamics in patients may indicate an underlying imbalance between stable and unstable brain states. Deviating patterns of EEG variability and other stability measures of brain activity further confirm these observations (Koshiyama et al., 2018; Foerster et al., 2021; Marques-Carneiro et al., 2021; Hecker et al., 2022; Huang et al., 2022). In summary, the present paradigm together with the identified physiological effects are a good starting point for further research about stability features of brain activity comparing patients with healthy controls. This will be one of the next steps on our agenda.

## Data availability statement

The raw data supporting the conclusions of this article will be made available by the authors upon request.

## Ethics statement

The studies involving human participants were reviewed and approved by the University of Freiburg, Germany. The patients/participants provided their written informed consent to participate in this study.

## Author contributions

EJ, LH, and JK contributed to the conception and design of the study. EJ and LH contributed to the data acquisition. MW, LH, JK, and AA contributed to the conception of the analysis. MW and LH contributed to the analysis of the data and wrote all analysis code. JK and MW wrote the first draft of the manuscript. LT and JK contributed to the acquisition of the funding. All authors contributed to manuscript revision, read, and approved the submitted version.

## Footnotes

1
https://surfer.nmr.mgh.harvard.edu/

2
https://mne.tools/
